# Value Configurations Associated with Artificial Intelligence Literacy Among Medical Students: Findings from NCA and fsQCA

**DOI:** 10.3390/bs16091458

**Published:** 2026-08-22

**Authors:** Huiying Liu, Jia Xue, Xuesong Shang, Wan Wang, Yuping Wang, Anqi Li, Hanxiao Cheng

**Affiliations:** 1 Mental Health Education Center, Zhengzhou University, Zhengzhou 450001, China; liuhy@zzu.edu.cn (H.L.); sxs@zzu.edu.cn (X.S.); wangwan@zzu.edu.cn (W.W.); wangyp@zzu.edu.cn (Y.W.); 2 School of Marxism, Zhengzhou University, 100 Science Avenue, Zhengzhou 450001, China; 3 School of Education, Zhengzhou University, 100 Science Avenue, Zhengzhou 450001, China; laq138@gs.zzu.edu.cn

**Keywords:** artificial intelligence literacy, medical students, healthcare education, value orientation, medical ethics

## Abstract

Artificial intelligence (AI) is increasingly integrated into healthcare education, clinical decision-making, and future practice. For medical students, AI literacy entails technical understanding, practical competence, ethical awareness, value-based judgment, and responsible engagement. This study examines how culturally embedded value orientations are associated with Chinese medical students’ perceived AI literacy, as assessed using a self-report instrument. In a cross-sectional sample of 1500 medical students enrolled at a comprehensive university in Henan Province, China, AI literacy was assessed using the 12-item Artificial Intelligence Literacy Scale (AILS), a self-report measure whose scores represented perceived AI literacy. Value orientations were measured using the 32-item Chinese Values Questionnaire (CVQ), comprising eight value dimensions. NCA and fsQCA were conducted to examine necessary conditions and configurational associations with membership in the high perceived AI literacy set. No single value dimension met the criterion for set-theoretic necessity with respect to membership in the high self-reported AI literacy set, and no individual condition met the fsQCA necessity consistency threshold of 0.90. Four sufficient configurations associated with high perceived AI literacy were identified, with an overall solution consistency of 0.867 and coverage of 0.383. Moral Self-Discipline and Public Interest repeatedly appeared as core or peripheral conditions. These results suggest that high perceived AI literacy was associated with multiple combinations of value orientations rather than with a single value dimension. High perceived AI literacy was associated with multiple value configurations rather than one dominant value orientation. Empirically, this study applies configurational analysis to understand how value orientations are associated with AI literacy, complementing existing research on knowledge, attitudes, and readiness. These context-bound associations may inform future research on whether medical AI curricula can integrate technical training with ethical reflection and public-oriented professional values.

## 1. Introduction

Artificial intelligence (AI) is increasingly integrated into healthcare, including diagnostic processes, decision support, and digital health systems (Alowais et al., 2023). AI technologies support medical imaging analysis, diagnostic classification, risk stratification, and clinical workflow efficiency (Liu et al., 2019; Rajkomar et al., 2018; Wenderott et al., 2024). Generative AI has further expanded applications in clinical documentation and clinical knowledge support (Shah et al., 2025; Singhal et al., 2023). As these technologies become embedded in routine clinical workflows, future healthcare professionals will inevitably practice in environments where human–AI collaboration is standard rather than exceptional (Gray et al., 2022).

Yet the implications of this transformation extend beyond healthcare practice, posing direct challenges to the goals and content of medical education. Medical students are increasingly exposed to AI-enabled tools in learning and clinical training. However, formal curricular preparation remains uneven and insufficient (McCoy et al., 2020; Wood et al., 2021). Existing studies show that many medical students recognize the importance of AI and express positive attitudes toward its future use, while also reporting limited knowledge, inadequate training, and uncertainty about how AI should be responsibly integrated into professional learning (Civaner et al., 2022; Jackson et al., 2024; Sami et al., 2025; Subaveerapandiyan et al., 2024).

Positive attitudes and frequent exposure do not necessarily indicate the capacity to use AI in a critically informed and professionally responsible manner (Jackson et al., 2024; Laupichler et al., 2024). Rather, current evidence indicates substantial engagement with AI alongside gaps in formal curricular preparation, leaving much AI-related learning self-directed, fragmented, and tool-oriented (Civaner et al., 2022; Wood et al., 2021). This mismatch raises concerns that students may use AI without first developing the conceptual, critical, and ethical capacities required to evaluate its outputs, recognize its limitations, and maintain appropriate human oversight (McCoy et al., 2020; Subaveerapandiyan et al., 2024). Against this background, value orientations become analytically relevant, as responsible AI use requires judgments about appropriate purposes, acceptable risks, protected interests, and human responsibility (Char et al., 2018; Li et al., 2023). These considerations are value-laden, involving decisions about what should be prioritized, justified, and safeguarded in AI-supported learning and future healthcare work (Masters, 2023; Zhang & Zhang, 2023). Nevertheless, limited attention has been given to how culturally embedded value orientations are associated with AI literacy among medical students.

From a value–behavior perspective, such judgments may be related to the goals individuals prioritize when interpreting and responding to value-relevant situations (Bardi & Schwartz, 2003). For medical students, this issue is particularly important because their use of AI is connected to the formation of professional judgment, patient-centered responsibility, and ethical awareness in healthcare education (Cooper & Rodman, 2023; McCoy et al., 2020). These concerns are consistent with examining medical students’ AI literacy as a value-relevant educational outcome rather than a purely technical competence (Ang, 2025). At the cross-sectional level, AI literacy may be associated with both AI-related learning experiences and students’ value-oriented interpretations of responsible AI use. Value orientations may be relevant to how students interpret the purposes, limits, and responsibilities of AI use (Gabriel, 2020; van de Poel, 2020). Rather than treating AI literacy as a purely knowledge-based outcome, research needs to consider the value standards that inform students’ understanding of responsible AI engagement. Existing studies have primarily focused on knowledge, attitudes, readiness, and intention to use AI (Boillat et al., 2022; Lugito et al., 2024; Si, 2025), while giving less attention to the value orientations associated with AI literacy among medical students.

In this study, Jin’s Contemporary Chinese Values framework is appropriate for examining the value orientations of Chinese medical students by measuring culturally specific goal orientations, including Wealth and Power, Family Orientation, Moral Self-Discipline, Law-Abiding Conformity, Practical Competence, Public Interest, Relational Affection, and Prestige Achievement (Jin et al., 2009). The eight dimensions encompass personal, relational, self-regulatory, practical, and public-oriented concerns. These concerns are relevant to how students evaluate AI outputs, judge appropriate use, weigh potential risks, and consider patient and public interests (Ang, 2025). Medical students who endorse different values may emphasize different considerations when evaluating AI use, including efficiency, accuracy, fairness, compliance, or patient welfare. The framework therefore allows this study to examine whether distinct value orientations are associated with different levels or configurations of AI literacy among Chinese medical students.

Given that these value orientations may coexist within individuals, their association with AI literacy may involve different combinations of values rather than a single isolated dimension. The present study combines Necessary Condition Analysis (NCA) and fuzzy-set Qualitative Comparative Analysis (fsQCA), thereby complementing the linear approaches commonly used in existing research. NCA identifies whether specific value orientations constitute necessary conditions for membership in the high AI literacy set (Dul, 2016; Dul et al., 2023), whereas fsQCA identifies combinations of values sufficient for membership in that set (Pappas & Woodside, 2021). This approach allows AI literacy to be examined as an outcome associated with multiple value-based configurations. This analytical strategy is appropriate for examining whether theoretically related value orientations coexist in configurations associated with AI literacy rather than only as isolated correlates.

Guided by the theoretical framework, we examined whether medical students’ value orientations were associated with their AI literacy. To this end, the study adopted a configurational approach to examine the association between value orientations and AI literacy among medical students. In this study, “sufficient” referred to set-theoretic sufficiency within calibrated fuzzy-set data and did not imply temporal precedence, experimental causation, or causal sufficiency. This study addressed two research questions:

Question 1: Which value orientations meet the criterion for set-theoretic necessity in relation to membership in the high AI literacy set among medical students?

Question 2: Which configurations of value orientations are sufficient, in the set-theoretic sense, for membership in the high AI literacy set among medical students?

## 2. Review of the Literature

### 2.1. AI Literacy Among Medical Students

AI literacy has received growing attention in medical education, where the use of AI is often discussed alongside clinical judgment, patient welfare, accountability, fairness, and appropriate human oversight. AI literacy is broadly understood as the competencies required to understand AI, interact effectively with AI systems, critically evaluate their outputs, and engage with them in an informed and ethical manner (Long & Magerko, 2020). In a related multidimensional formulation, Wang et al. (2023) conceptualized AI literacy in terms of awareness, usage, evaluation, and ethics. Recent studies involving medical and nursing students have drawn on the definition and measure developed by Wang et al. (2023). These studies have examined AI literacy in relation to such factors as readiness for medical AI, attitudes toward AI, and prior exposure to and use of AI technologies (Boztepe et al., 2025; Catan-Inan, 2026; Lan et al., 2026). Evidence from these samples supports the relevance of this definition to health professions education. The use of the corresponding instrument among medical and nursing students offers preliminary evidence of its suitability for assessing AI literacy among students in the health professions.

### 2.2. Value Dimensions in Medical Education and AI Engagement

Values may be associated with how individuals interpret ambiguous situations, prioritize competing concerns, and evaluate alternative courses of action (Maio, 2010; Sagiv & Roccas, 2021; Schwartz, 2012). They are defined as stable, trans-situational motivational goals guiding evaluation, preference, and action (Maio, 2010; Schwartz, 2015). They shape what individuals regard as important and desirable, and they influence responses to professional demands and emerging technologies (Sagiv & Roccas, 2021). Unlike temporary preferences or situational attitudes, values provide relatively enduring standards for judging what should be prioritized and what should be constrained (Schwartz, 2012). Values guide what individuals attend to, how they interpret situations, how they evaluate possible actions, and which goals they pursue (Sagiv & Roccas, 2021). Within the Contemporary Chinese Values framework, Wealth and Power and Prestige Achievement reflect personal advancement and social recognition, whereas Family Orientation and Relational Affection concern familial and interpersonal commitments. Moral Self-Discipline and Law-Abiding Conformity reflect self-regulation and adherence to social norms, while Practical Competence concerns practical accomplishment and Public Interest concerns collective welfare and broader social responsibility. These value orientations are conceptually relevant to how students evaluate AI outputs, judge appropriate use, weigh potential risks, and consider patient and public interests.

In the context of AI-integrated healthcare, these value orientations may be relevant to how students scrutinize AI recommendations, protect patient interests, respect the limits of technological authority, and maintain accountability for clinical decisions (Char et al., 2018; Li et al., 2023; Varkey, 2020). Recent discussions of humanistic care in AI-enabled medical education provide a conceptual basis for examining whether Relational Affection and Public Interest are associated with attention to patient dignity, communication, and the potential dehumanizing effects of technology (Maharem, 2024; Simou, 2025; Sivarajah et al., 2023). Recent studies have linked medical students’ familiarity with AI to their attitudes and intentions regarding its application (Duan et al., 2025; Sami et al., 2025). Within the CVQ framework, this evidence provides a conceptual basis for examining Practical Competence in relation to perceived AI literacy. Moreover, individuals with stronger self-regulatory orientations may be better positioned to avoid over-reliance and maintain critical engagement during AI-assisted learning (Shi et al., 2025; Zhai et al., 2024).

### 2.3. Embedding Values in AI Literacy Among Medical Students

The value–behavior relations perspective provides a framework for examining how value orientations may be associated with perceived AI literacy and how such associations may appear. Bardi and Schwartz (2003) posited that individuals are more inclined to enact behaviors that facilitate the attainment of highly prioritized values. Subsequent research suggests that the association between values and behavior may be stronger when values are highly endorsed and personally important to the individual, and when the behavioral domain is perceived as value-relevant (Lee et al., 2022). AI use in healthcare should be understood as a normative practice rather than a purely technical intervention. Accordingly, value orientations may be particularly relevant when students perceive technological decisions as intertwined with their professional commitments.

Notably, AI literacy may be considered a value-relevant domain in which students’ engagement with technology may be associated with their underlying motivational goals. Applied to healthcare education, perceived AI literacy may therefore be associated not only with exposure to AI-related knowledge but also with students’ value-based interpretations of responsible AI use (Char et al., 2018; Masters, 2023; van de Poel, 2020). Consistent with the value–behavior framework, these subjective interpretations may be reflected in how students report their engagement with AI, particularly in relation to the cultural and ethical priorities they endorse. Responsible AI engagement may co-occur with moral restraint, public-interest concern, practical competence, and humanistic sensitivity. These orientations may form part of configurations associated with perceived AI literacy. The extent to which these value configurations are reflected in subsequent AI-related practices remains to be established through longitudinal research conducted before and during clinical practice.

## 3. Methods

### 3.1. Study Design

Data for this study were drawn from the medical student component of a larger cross-disciplinary comparative survey conducted by the research team. The present analysis focused exclusively on medical students. A cross-sectional online survey was administered at a comprehensive university in Henan Province, China, between 26 September and 7 October 2025. Participation was voluntary, and electronic informed consent was obtained before questionnaire completion. Upon completion, participants received a small nonmonetary token of appreciation, such as a pen or notebook, valued at no more than RMB 2. Of the 9600 medical students invited to participate, 8737 submitted a questionnaire, yielding a response rate of 91.0%. Eligible respondents were aged 18–65 years. Records were excluded because age fell outside the specified range (n = 321), completion time was less than 300 s (n = 31), or the maximum response option was selected for all 12 AILS items (n = 193). The 300 s threshold was established a priori to allow sufficient time to complete the full questionnaire package, which included the AILS (Wang et al., 2023), the CVQ (Jin et al., 2009), and supplementary measures from the broader project. In the AILS, Items 2, 5, and 11 require reverse scoring. Selecting the maximum option for all 12 items simultaneously endorsed oppositely worded statements and, after recoding, yielded scores of 1 for these three items and 10 for all remaining items. Records exhibiting this internally inconsistent pattern were excluded under a predefined data quality criterion before random sampling and analysis. After the eligibility and data quality criteria were applied, 8192 records remained in the sampling frame of the parent project. In accordance with the project’s data access procedures, authorized data managers selected 1500 deidentified records using proportionate stratified random sampling without replacement. The strata were defined by gender, academic grade, and academic level (undergraduate or postgraduate). The resulting dataset constituted the analytic sample for the present study.

### 3.2. Participants and Data Collection

The study population comprised medical students enrolled at the medical school of one comprehensive university in Henan Province, China, who were admitted between 2021 and 2025. The study did not represent medical students from universities across Henan Province. Recruitment was conducted through classroom-based invitations at the participating medical school. Trained members of the research team introduced the study and distributed the online questionnaire link or QR code to eligible students. Before accessing the questionnaire items, participants reviewed an electronic information statement and indicated informed consent by selecting the consent option. The survey platform restricted each authenticated participant’s account to a single submission. The exported dataset was also screened for exact duplicate records during data cleaning, and none were identified. The final analytic sample comprised 1500 medical students randomly selected without replacement from the pre-specified eligible sampling frame through proportionate stratified sampling. The sample ranged from 18 to 35 years (*M* = 21.86, *SD* = 3.05). Of these, 865 (57.67%) were female and 635 (42.33%) were male. Regarding academic stage, 418 students (27.9%) were in their first or second year of undergraduate study; 447 students (29.8%) were in their third, fourth, or fifth year of undergraduate study, and 635 students (42.3%) were postgraduate students.

### 3.3. Measures

Value orientations were assessed using the Chinese Values Questionnaire (CVQ) developed by Jin et al. (2009). The CVQ is a 32-item instrument measuring eight dimensions, including Wealth and Power, Family Orientation, Moral Self-Discipline, Law-Abiding Conformity, Practical Competence, Public Interest, Relational Affection, and Prestige Achievement. Responses were recorded on a six-point Likert scale ranging from 1 (“strongly disagree”) to 6 (“strongly agree”). Dimension scores were calculated by averaging the corresponding item responses, with higher scores indicating stronger endorsement of each value orientation. The validated Chinese version was administered. In the present sample, the total CVQ score demonstrated good internal consistency (Cronbach’s *α* = 0.899). Cronbach’s *α* was 0.829 for Wealth and Power, 0.441 for Family Orientation, 0.759 for Moral Self-Discipline, 0.646 for Law-Abiding Conformity, 0.724 for Practical Competence, 0.801 for Public Interest, 0.590 for Relational Affection, and 0.660 for Prestige Achievement.

Participants’ AI literacy was assessed using the Chinese version of the 12-item Artificial Intelligence Literacy Scale (AILS) developed by Wang et al. (2023). The AILS is a self-report instrument that measures respondents’ perceptions of their AI literacy rather than objectively demonstrated knowledge or performance. It comprises four dimensions: Awareness, Usage, Evaluation, and Ethics. The scale was independently translated into Chinese by two bilingual scholars, reviewed for linguistic clarity and cultural relevance by two Chinese-language experts, and independently back-translated by two English-language experts. Comparison of the original and back-translated versions supported conceptual consistency, and no items were removed. Items were rated on a 10-point Likert scale ranging from 1 (“strongly disagree”) to 10 (“strongly agree”), with higher scores indicating higher levels of self-perceived AI literacy. Items 2, 5, and 11 were reverse-coded in accordance with the original scoring instructions before the total and dimension scores were calculated.

The total AILS score demonstrated good internal consistency in the present sample (Cronbach’s α = 0.877). Cronbach’s α was 0.489 for Awareness, 0.607 for Usage, 0.939 for Evaluation, and 0.553 for Ethics. Confirmatory factor analysis of the correlated four-factor model yielded χ^2^(48) = 651.10, *p* < 0.001, CFI = 0.921, TLI = 0.892, RMSEA = 0.117, 90% CI [0.109, 0.125], and SRMR = 0.079. Reliability and convergent validity varied across the four dimensions. Only Usage and Evaluation met the conventional reference values for both composite reliability and average variance extracted. Evidence of discriminant validity was mixed. As a general self-report measure, the AILS does not assess objectively demonstrated performance or healthcare-specific competencies, such as clinical application, patient safety, and professional accountability. The total score was retained as the outcome based on the adequate internal consistency of the overall scale (α = 0.877) and its aggregation of items across all four subdimensions. The mixed fit of the four-factor model and the weak reliability of some subdimensions concern dimension-level structure rather than the aggregate score. In the subsequent configurational analyses, the total AILS score served as the observed outcome and was interpreted as an indicator of perceived AI literacy.

Socio-demographic information, including age, gender, academic level (undergraduate or postgraduate), and year of enrollment, was also collected to describe the sample.

### 3.4. Statistical Analysis

Data were analyzed using SPSS (Version 27.0) for correlation analysis, R (Version 4.5.1) for necessary condition analysis (NCA), and fsQCA software (Version 4.1) for fuzzy-set qualitative comparative analysis. These analyses examined relationships between the eight CVQ value dimensions (conditions) and perceived AI literacy measured by the AILS (outcome). First, NCA assessed necessity using ceiling-based effect sizes and bottleneck levels. Conditions with effect sizes below 0.10 were not interpreted as substantively necessary. Statistical significance was treated as supplementary evidence and did not override a negligible effect size (Dul, 2016; Dul et al., 2020). Subsequently, fsQCA was used to identify configurations sufficient for membership in the high AI literacy set based on self-reported AILS scores. Variables were calibrated using the direct method. The 90th, 50th, and 10th percentiles were used as the full-membership, crossover, and full non-membership anchors, respectively. Because externally validated cut points are unavailable for the AILS and CVQ in this population, these percentile anchors were not interpreted as theory-derived or clinical thresholds. They operationalized relative set membership within the participating sample. Sensitivity to this distribution-based calibration was assessed using alternative anchors at the 85th, 50th, and 15th percentiles (Fiss, 2011; Greckhamer et al., 2018; Pappas & Woodside, 2021; Ragin, 2008). A constant of 0.001 was added to calibrated scores of exactly 0.5 to avoid ambiguity at the crossover point (Cangialosi, 2023; Fiss, 2011). The calibration thresholds for all conditions and the outcome are presented in Table 1.

A truth table was constructed using a frequency cutoff of 10, a raw consistency threshold of 0.80, and a proportional reduction in inconsistency (PRI) threshold of 0.65. Among the truth-table rows satisfying the frequency and PRI requirements, the lowest raw consistency retained for sufficiency analysis was 0.896 (Greckhamer et al., 2018; Pappas & Woodside, 2021). No a priori directional expectations were specified, and no logical remainder was treated as an easy counterfactual. Under this specification, the intermediate solution was identical to the complex solution, whereas the parsimonious solution permitted all logical remainders. The resulting solutions were interpreted descriptively rather than as theory-guided causal models.

Robustness was assessed by varying the calibration anchors, frequency cutoff, PRI threshold, and raw consistency threshold while holding all other specifications constant. The alternative calibration used the 85th, 50th, and 15th percentiles. The frequency cutoff was varied from 10 to 5 and 15, the PRI threshold from 0.65 to 0.60 and 0.70, and the raw consistency threshold from 0.80 to 0.85. Configurational stability was evaluated by comparing the number of retained configurations, overall solution coverage, and overall solution consistency across specifications (Greckhamer et al., 2018; Oana & Schneider, 2024).

## 4. Results

### 4.1. Common Method Bias

Harman’s single-factor test was conducted as a preliminary diagnostic of potential common method variance. Seven factors with eigenvalues greater than 1 were extracted, and the first unrotated factor accounted for only 24.25% of the total variance, which is well below the commonly used 50% threshold. This result is only preliminary evidence that a single general factor was not dominant. Because the substantive measures were collected through a single self-report questionnaire at one time point, residual common method variance cannot be ruled out (Aguirre-Urreta & Hu, 2019; Podsakoff et al., 2024).

### 4.2. Descriptive Statistics and Correlations

Descriptive statistics and Spearman’s rank correlations among the study variables are presented in Table S1 in the Supplementary Materials. Demographic variables showed limited bivariate associations with the total AILS score. The total AILS score was positively correlated with Practical Competence (r = 0.368, *p* < 0.001), Public Interest (r = 0.422, *p* < 0.001), and Moral Self-Discipline (r = 0.315, *p* < 0.001), and showed weaker positive correlations with Law-Abiding Conformity (r = 0.113, *p* < 0.001) and Relational Affection (r = 0.121, *p* < 0.001). No statistically significant bivariate correlations were observed for Wealth and Power, Family Orientation, or Prestige Achievement. These correlations were reported for descriptive context only. In fsQCA, the relevance of a condition is evaluated through set-theoretic necessity and sufficiency rather than through its pairwise correlation with the outcome. Accordingly, a non-significant bivariate correlation does not imply that a condition is irrelevant to a configuration.

### 4.3. Analysis of Necessary Conditions

The fsQCA analysis first examined whether any antecedent condition or its logical negation was necessary for membership in the high perceived AI literacy set. Within fsQCA, a condition is treated as necessary when its set-theoretic consistency exceeds 0.90 (Schneider & Wagemann, 2012). As shown in Table 2, none of the consistency values reached this threshold. Practical Competence and Public Interest showed the highest consistency values, but both remained below 0.90. Thus, neither the presence nor the absence of any single value dimension met the pre-specified consistency threshold for set-theoretic necessity with respect to membership in the high AI literacy set indicated by self-reported scores.

To complement the fsQCA necessity analysis, NCA was applied to the same calibrated condition and outcome scores as an additional assessment of necessity. The two approaches address related but distinct questions. fsQCA evaluates set-theoretic necessity through consistency, whereas NCA estimates the extent to which a condition constrains the outcome space and quantifies this constraint using an effect size. NCA therefore examines necessary-but-not-sufficient relations and does not replace the configurational analysis of sufficiency conducted by fsQCA. The use of calibrated fuzzy-set scores kept the condition and outcome definitions aligned across the two analyses. This approach is suitable for examining necessary-but-not-sufficient relationships because it assesses whether an outcome is constrained by the absence of a minimum level of a given condition (Dul, 2016; Vis & Dul, 2018). As shown in Table 3, the effect sizes (*d*) of all eight value dimensions were below the recommended threshold of 0.10 under both ceiling regression and ceiling envelopment estimation. Although some permutation tests were statistically significant, the corresponding effect sizes were zero or negligible. Accordingly, no value dimension was interpreted as a substantively necessary condition. Within the calibrated sample, membership in the high perceived AI literacy set was not consistently bounded by a substantively meaningful minimum level of any single value dimension. This finding supported the decision to examine combinations of value dimensions rather than isolated necessary conditions. It suggests that no single dominant value orientation adequately characterized high perceived AI literacy in this sample. This result is consistent with configurational research in which complex outcomes are examined in relation to combinations of conditions rather than isolated factors (Fiss, 2011; Furnari et al., 2021).

The bottleneck analysis further supported this result (Table 4). From 0% to 90% of the outcome level, all eight value dimensions were marked as “NN” (Not Necessary). Only at the extreme 100% outcome level did several small numerical bottleneck values appear (Dul et al., 2023). At this level, the estimated bottleneck values were 1.0% for Wealth and Power, 1.0% for Family Orientation, 1.9% for Moral Self-Discipline, 8.4% for Practical Competence, and 10.9% for Public Interest. Because these bottleneck estimates appeared only at the extreme 100% outcome level and were accompanied by negligible NCA effect sizes, they should be treated as descriptive boundary estimates rather than as substantively meaningful thresholds. These findings should not be interpreted as showing that values are irrelevant to self-reported AI literacy. Rather, they indicate that no single value dimension functions as an isolated prerequisite within the calibrated sample.

### 4.4. Truth Table Construction and Configuration Analysis

The initial truth table was generated using fsQCA 4.1. To filter configurations lacking sufficient explanatory power, thresholds were set for case frequency (≥10), raw consistency (≥0.80), and PRI consistency (≥0.65) (Douglas et al., 2020; Pappas & Woodside, 2021). The fsQCA typically yields complex, intermediate, and parsimonious solutions. Four minimum parsimonious models were obtained. Model M2 was selected from the fsQCA 4.1 prime implicant chart and served as the reference for distinguishing core from peripheral conditions. A condition was classified as core when it appeared in an intermediate solution term and in the corresponding M2 parsimonious term or terms. Conditions present only in the intermediate term were classified as peripheral (Fiss, 2011). The complete truth table and the complex, intermediate, and parsimonious solutions are reported in Tables S2–S5 of the Supplementary Materials. The intermediate solution comprised four set-theoretically sufficient configurations associated with membership in the high AI literacy set indicated by self-reported scores. Table 5 reports the core and peripheral conditions, raw coverage, unique coverage, and consistency of each configuration. These configurations represent set-theoretically sufficient patterns within the calibrated sample and should not be interpreted as temporal or causal pathways.

#### Configurations of High AI Literacy

As shown in Table 5, the intermediate solution identified four set-theoretically sufficient configurations associated with membership in the high AI literacy set indicated by self-reported scores. The overall solution consistency was 0.867, and the overall coverage was 0.383. The retained configurations accounted for 38.3% of membership in the outcome set. A substantial share of outcome set membership remained outside the coverage of the retained configurations. In fsQCA, coverage represents empirical relevance within calibrated data rather than explained variance in regression analysis. Coverage should be interpreted as partial rather than comprehensive (Fiss, 2011). Equifinality was evident across the identified configurations. No single configuration accounted for all membership in the high self-reported AI literacy set. Instead, several distinct combinations of antecedent conditions were associated with membership in this outcome set. Moral Self-Discipline was retained as a core condition across all four configurations. Public Interest also recurred throughout the solution. It occupied a core position in C4 and a peripheral position in C1, C2 and C3. The recurring presence of these two conditions was associated with higher self-reported AI literacy across the identified configurations. These results do not support inferences about causal or universal mechanisms. In the configuration descriptions, presence and absence denote calibrated set membership relative to the specified anchors. Non-membership in a condition should not be interpreted as indicating that respondents entirely lacked or rejected the corresponding value orientation. It denotes relatively low membership in the calibrated set under the specified anchors.

Configuration 1 (C1) may be described as an “autonomous ethical” configuration, with raw coverage of 0.194 and consistency of 0.904. This configuration is characterized by the core presence of moral self-Discipline, together with the core absence of Family Orientation, Law-Abiding Conformity, and Prestige Achievement. It also includes the peripheral presence of Public Interest and the peripheral absence of Wealth and Power and Relational Affection. This pattern is associated with AI literacy configurations characterized by the recurrent presence of Moral Self-Discipline. Family Orientation, Prestige Achievement, and Law-Abiding Conformity are less prominent across the identified configurations. The apparent absence of Family Orientation reflects low calibrated membership rather than a genuine lack of this value, and may partly stem from measurement imprecision in this dimension.

Configuration 2 (C2) may be described as a “resource-supported, nonconformity-oriented” configuration, with raw coverage of 0.170 and the highest consistency among the four configurations (0.912). C2 is defined by the core presence of Moral Self-Discipline and the core absence of Law-Abiding Conformity. It also includes the peripheral presence of Wealth and Power, Family Orientation, Practical Competence, Public Interest, Relational Affection, and Prestige Achievement. This configuration is characterized by the coexistence of ethical self-regulation with practical, interpersonal, and public-oriented value conditions in the high perceived AI literacy set.

Configuration 3 (C3) may be described as a “pragmatic public-interest” configuration, with raw coverage of 0.248 and consistency of 0.908. Its core conditions were Moral Self-Discipline and low membership in Family Orientation and Prestige Achievement. Practical Competence and Public Interest, together with low membership in Wealth and Power, were peripheral conditions. C3 therefore combines ethical self-regulation with values oriented toward practical competence and public interest in relation to high perceived AI literacy.

Configuration 4 (C4) may be described as a “relational and community-oriented” configuration. It had the highest raw coverage of 0.254 and unique coverage of 0.055, with consistency of 0.888. The core conditions are Moral Self-Discipline, Public Interest, and Relational Affection, combined with the core absence of Wealth and Power. Peripheral conditions include Law-Abiding Conformity and Practical Competence. This configuration is characterized by the coexistence of relational, public-oriented, and ethically self-regulatory value conditions within the high AI literacy set based on self-reported scores.

### 4.5. Robustness Test

Robustness was assessed by varying the calibration anchors, frequency cutoff, PRI threshold, and raw consistency threshold, as reported in Table 6. The original four configurations were reproduced under the stricter consistency threshold. A frequency cutoff of 15 also yielded four configurations, although their detailed composition varied. Greater variation emerged under the lower frequency cutoff, the higher PRI threshold, and the alternative calibration specification. Moral Self-Discipline remained present in most alternative solutions. Overall, the results were stable under the stricter consistency threshold but were sensitive to the calibration specification, the lower frequency cutoff, and the PRI threshold. Table S6 in the Supplementary Materials reports the configurations retained under each specification together with the core or peripheral status of every condition. Across the 32 retained configurations, Moral Self-Discipline was present in 30 (94%) and Public Interest in 29 (91%); neither condition entered any retained configuration in negated form, and at least one of the two was present in every retained configuration.

## 5. Discussion

### 5.1. Principal Findings

**Figure 1:**
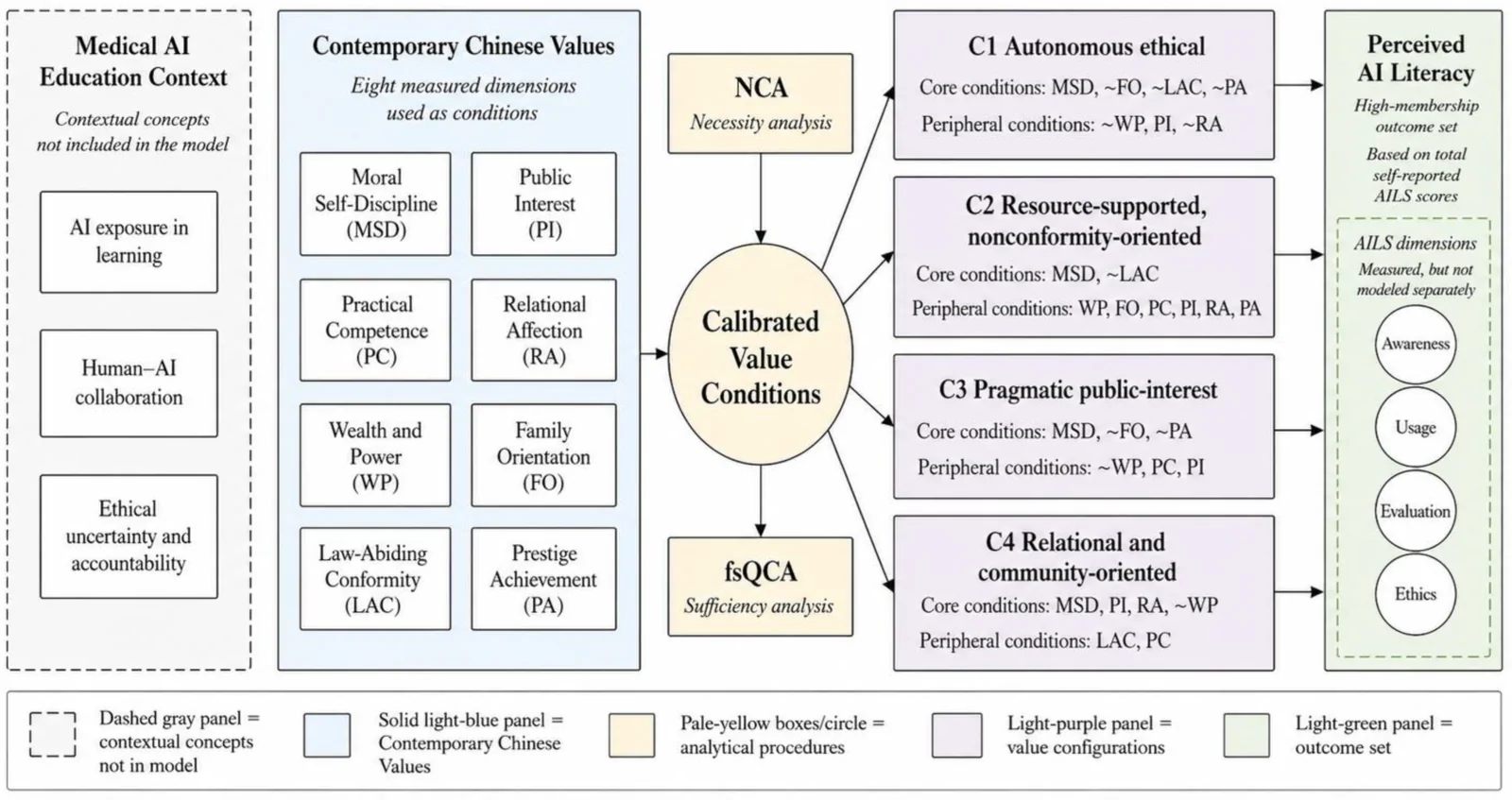
Conceptual framework for the configurational analysis. Note. AILS, Artificial Intelligence Literacy Scale; NCA, necessary condition analysis; fsQCA, fuzzy-set qualitative comparative analysis; WP, Wealth and Power; FO, Family Orientation; MSD, Moral Self-Discipline; LAC, Law-Abiding Conformity; PC, Practical Competence; PI, Public Interest; RA, Relational Affection; PA, Prestige Achievement. The dashed panel provides conceptual context and was not included in the configurational model. The outcome was based on the total self-reported AILS score rather than its four separate dimensions. The tilde “~” denotes set negation, indicating low membership in the corresponding calibrated set. Arrows represent the analytical structure only, and the configuration names are interpretive labels.

The empirical contribution of this study lies in examining medical students’ perceived AI literacy from a configurational perspective rather than by considering value dimensions in isolation. Existing AI literacy frameworks have mainly clarified what learners should know and be able to do, such as understanding AI concepts, using tools, evaluating outputs, and recognizing ethical issues (Long & Magerko, 2020; Ng et al., 2021). The present study provides a context-specific examination of how moral, practical, public-oriented, and relational value orientations co-occur in configurations associated with perceived AI literacy among medical students. In this sense, the results describe how different value orientations co-occur in configurations associated with perceived AI literacy in AI-supported medical learning. Figure 1 summarizes the four value configurations observed in association with high AI literacy set based on self-reported AILS scores in this sample.

#### 5.1.1. The Configurational Nature of AI Literacy Among Medical Students

This study addressed two questions raised in the introduction. The first was whether any specific value orientation constituted a necessary condition for high AI literacy among medical students. The second concerned which configurations of value orientations were sufficient for membership in the high perceived AI literacy set. These questions were grounded in a configurational logic, which assumes that an outcome may arise from combinations of conditions rather than from the isolated effect of a single factor (Dul, 2016; Fiss, 2011; Schneider & Wagemann, 2012). The present findings were consistent with this configurational view at the set-theoretic level. No single value dimension was found to be indispensable for high AI literacy. Instead, high perceived AI literacy was associated with several sufficient value configurations.

The fsQCA identified four set-theoretically sufficient configurations, a pattern consistent with equifinality. Across the four configurations, Moral Self-Discipline and Public Interest orientation appeared repeatedly as core or peripheral conditions. This finding is consistent with an empirical application of configurational analysis to the study of perceived AI literacy. It suggests that perceived AI literacy in medical education may coexist with cognitive, ethical, professional, and motivational orientations in different combinations. In this respect, the study offers a context-specific empirical application of value–behavior reasoning to perceived AI literacy in professional education (Lee et al., 2022; Sagiv & Roccas, 2021; Schwartz, 2015).

At an interpretive level, the four configurations suggest a layered conceptual pattern. Moral Self-Discipline may represent internal self-regulation relevant to cautious AI use and critical judgment. Public Interest may reflect attention to patient welfare and public health. Practical Competence may correspond to an emphasis on effective tool use. Relational Affection is theoretically relevant to the interpersonal aspects of AI engagement, including communication, empathy, and collaborative learning. These interpretations are theoretically informed. They were not directly tested as temporal or psychological mechanisms. Taken together, the findings are consistent with a value-based interpretation of perceived AI literacy as a professional capacity that extends beyond discrete technical skills.

#### 5.1.2. The Centrality of Moral Self-Discipline and Public Interest Orientation

The recurrent presence of Moral Self-Discipline across all four configurations merits cautious interpretation. In this sample, it was consistently associated with membership in the high perceived AI literacy set when combined with other value conditions. This interpretation is compatible with theoretical perspectives that describe values as enduring standards for judging what should be prioritized and constrained (Lee et al., 2022). The cross-sectional data do not establish that Moral Self-Discipline enables critical evaluation or constitutes a foundation for responsible AI use.

Equally significant is the consistent role of public interest orientation. Medical students are not merely learners acquiring technical skills; they are future physicians whose engagement with technology must remain subordinate to patient welfare, public health, and professional accountability. The recurrence of public interest orientation across the configurations was consistent with the relevance of medicine’s social mission to students’ perceived AI literacy. This finding echoes the argument proposed by Wartman and Combs in “Medical Education Must Move from the Information Age to the Age of Artificial Intelligence”, that medical education must respond to AI not only by adding information technology content but also by preparing physicians for a transformed professional environment (Wartman & Combs, 2018).

This interpretation also challenges a narrow technology-acceptance or readiness logic. Although classical technology acceptance theories have been highly influential, they mainly explain technology use through users’ instrumental evaluations, behavioral intentions, and facilitating conditions. The Technology Acceptance Model emphasizes perceived usefulness and perceived ease of use as key determinants of user acceptance (Davis, 1989), whereas the Unified Theory of Acceptance and Use of Technology integrates multiple acceptance models into a unified framework comprising performance expectancy, effort expectancy, social influence, facilitating conditions, and behavioral intention (Venkatesh et al., 2003). These models are useful for explaining whether individuals are likely to adopt technology, but they give limited attention to the value-based motivations that may shape responsible AI engagement. In medical education, the decisive question is not only whether students are willing or able to use AI, but whether they can judge when AI use is ethically appropriate, whose interests it serves, and how human accountability should be preserved. In the present sample, Moral Self-Discipline and Public Interest were recurrently present in configurations characterized by higher perceived AI literacy. The data do not establish that these orientations convert technical capability into professional literacy.

The weaker role of Wealth and Power and Prestige Achievement is also theoretically interpretable. These orientations conceptually correspond to Schwartz’s (1992) self-enhancement values, which emphasize personal success, social status, dominance, and control over resources. Although such values may motivate instrumental engagement with AI, they are less directly aligned with the evaluative and ethical components of AI literacy. They may encourage instrumental or status-oriented engagement with AI, but they do not necessarily promote critical evaluation, ethical caution, or patient-centered responsibility. Wealth and Power and Prestige Achievement were less recurrent in the retained configurations. In fuzzy-set terms, their absence denotes lower calibrated membership rather than a psychological lack of these values. The present data do not establish whether these orientations promote or impede critical, ethical, or patient-centered AI use.

#### 5.1.3. Methodological Contribution of Integrating NCA and fsQCA

Methodologically, the joint use of NCA and fsQCA provided a complementary set-theoretic perspective alongside conventional correlational analysis. These approaches address distinct analytical questions. NCA first tests whether any value dimension functions as a necessary bottleneck, while fsQCA then identifies configurations sufficient for membership in the outcome set within the calibrated data. The absence of a single necessary value condition, together with the presence of four sufficient configurations, supports the examination of conjunctural patterns rather than isolated conditions. Similar levels of perceived AI literacy may be observed among Chinese medical students with different combinations of value orientations. The set-theoretic relevance of a value may vary across configurations (Fiss, 2011; Greckhamer et al., 2018; Pappas & Woodside, 2021).

The findings are also consistent with the broader configurational literature, which characterizes complex educational and organizational outcomes in terms of conjunctural relations, equifinality, and configurational asymmetry rather than isolated net effects. Studies employing configurational theorizing have similarly shown that multiple configurations may be associated with the same outcome and that core conditions may be relevant only within specific combinations (Furnari et al., 2021; Misangyi et al., 2017). By applying this logic to perceived AI literacy in medical education, the present study provides a context-specific empirical application of configurational methods. These findings suggest that future research may examine perceived AI literacy as a context-sensitive phenomenon involving multiple value configurations and intersecting technical, ethical, and humanistic considerations. The robustness checks reported in Table 6 show that the number and composition of the configurations were not invariant. They shifted when the calibration anchors, the frequency cutoff, and the PRI threshold were changed. The four configurations were reproduced under the stricter consistency threshold and under the frequency cutoff of 15. The detailed composition, however, varied under the lower frequency cutoff, the higher PRI threshold, and the alternative calibration specification. This sensitivity is inherent to set-theoretic methods. It means that the reported configurations are contingent on the chosen analytical specifications. Among the conditions, Moral Self-Discipline was the most stable across the alternative specifications. Its stability supports interpretation of this condition as a robust element of the pattern.

### 5.2. Implications

The findings offer several tentative implications for medical AI education and healthcare practice. Given the cross-sectional, self-report design, these implications should be regarded as propositions for future curriculum research rather than as direct educational prescriptions. First, medical AI education may consider incorporating value-based reflection into AI literacy training. Rather than treating AI literacy as a purely technical competency, curricula could encourage students to consider how AI use relates to ethical judgment, patient welfare, public health, and professional responsibility. Case-based discussions and reflective exercises could be considered as educational approaches for integrating technical competence with responsible professional practice.

Notably, membership in the high perceived AI literacy set was associated with different value configurations. This observation may inform hypotheses for curriculum research, although the pedagogical relevance of these configurations requires evaluation in intervention studies. Future studies could examine whether activities emphasizing critical evaluation and ethical reflection are appropriate across different value orientations. Future intervention studies could test whether learning activities centered on critical evaluation, ethical reflection, clinical application, public health, or collaborative learning have differential effects across student groups. Such differentiated approaches remain hypotheses for curriculum research and require validation before they are used to define pedagogical profiles. In this sense, configurational analysis may offer one perspective for considering differentiated AI literacy education without assuming that all students must follow the same developmental route.

More broadly, the recurrent role of Moral Self-Discipline and Public Interest orientation suggests that perceived AI literacy in this sample was not represented solely by access to AI tools or technical proficiency. Medical schools and healthcare organizations may consider fostering educational environments in which AI is examined in relation to patient care and public health rather than presented as a technology for uncritical adoption. Professional development initiatives could accordingly emphasize accountability, appropriate human oversight, and the alignment of AI use with broader healthcare values.

### 5.3. Limitations

Several limitations should be noted. First, the cross-sectional design precludes causal inference and does not clarify the temporal ordering between value orientations and perceived AI literacy. Second, all measures were self-reported, which raises the possibility of social desirability bias and common method variance. Although Harman’s single-factor test provided a preliminary diagnostic, residual common method variance cannot be excluded. Third, the sample was drawn from a single university in China, and the identified configurations may reflect the cultural and educational context of that setting. Their transferability to medical students in other countries and educational systems therefore remains uncertain.

A further limitation concerns measurement. The AILS captures the general dimensions of Awareness, Usage, Evaluation, and Ethics, but may not fully represent the healthcare-specific competencies encompassed by the broader conceptualization of medical AI literacy. Clinical judgment, patient safety, professional accountability, and human oversight may therefore remain insufficiently assessed. The total CVQ and AILS scores showed good internal consistency. Several dimensions showed weak internal consistency. The lowest coefficients were observed for Family Orientation (α = 0.441), Awareness (α = 0.489), and Ethics (α = 0.553). Confirmatory factor analysis of the AILS model with four correlated factors produced mixed evidence of model fit. The CFI (0.921) and SRMR (0.079) suggested comparatively favorable fit. The TLI (0.892) and RMSEA (0.117) were less satisfactory. Family Orientation was included as a separate fsQCA condition despite its low reliability. Measurement imprecision in this dimension may have affected both its calibrated set membership and its apparent role within the identified configurations. The weak reliability of the Awareness and Ethics dimensions does not directly characterize the reliability of the total AILS outcome. It limits interpretation of the scale’s multidimensional structure. These measurement limitations should be considered when interpreting findings for individual dimensions and the composition of the identified configurations. Finally, the configurational model included only value orientations and did not incorporate other potentially relevant personal and contextual conditions, including prior AI exposure, digital competence, curriculum experience, clinical experience, institutional support, trust in AI, and access to AI tools. The omission of these variables may have influenced the identified configuration structure, consistency, and coverage. The resulting configurations should therefore be interpreted as value-oriented patterns within the specified model rather than as a comprehensive account of the conditions associated with perceived AI literacy. Future studies should integrate personal, educational, institutional, and technological conditions into broader configurational models. Longitudinal, multisite, and cross-national research using performance-based designs and validated healthcare-specific instruments is also needed.

## 6. Conclusions

This study examined configurational associations between contemporary Chinese value orientations and Chinese medical students’ perceived AI literacy using NCA and fsQCA. The configurational model was deliberately restricted to value orientations and did not incorporate other potentially relevant conditions associated with perceived AI literacy. Consequently, the identified configurations should not be regarded as an exhaustive account of the conditions associated with perceived AI literacy. The findings indicate that high levels of perceived AI literacy are associated with multiple set-theoretically sufficient configurations of value orientations rather than any single necessary value orientation. Moral Self-Discipline and Public Interest orientation appeared repeatedly as core or peripheral conditions in the identified configurations. Their recurrence was consistent with an association between ethical self-regulation, public-oriented values, and perceived AI literacy in this sample. In comparison with prior value–behavior studies focused primarily on discrete behavioral outcomes, this study provides a context-specific empirical application of configurational analysis to perceived AI literacy in medical education. Given the culturally specific nature of the Contemporary Chinese Values framework and the single-university Chinese sample, the identified configurations should be interpreted as context-bound associations. The reported configurations are also contingent on the analytical specifications used in the fsQCA procedure. The robustness checks indicated that their number and composition varied with the calibration anchors, the frequency cutoff, and the PRI threshold. The solution obtained in this study is therefore one representation that depends on the chosen specifications, rather than a unique or fixed result.

Within the scope of the value-oriented model examined here, these findings may inform discussions of AI literacy education that integrate technical training with ethical reasoning, critical evaluation of AI outputs, patient welfare, and professional responsibility. Within this sample, higher levels of perceived AI literacy were associated with different combinations of moral, practical, public-oriented, and relational values. These patterns may inform hypotheses for future curriculum development that integrates technical training with ethical reasoning, critical evaluation of AI outputs, patient welfare, and professional responsibility. Future intervention research could examine the educational value of such approaches across diverse medical education settings. Curricular initiatives could consider integrating technical competence with ethical reflection on responsible AI use.

## Figures and Tables

**Table 1:** Data calibration.

Conditions and Outcome	Full Membership	Crossover Point	Full Non-Membership
AI literacy	8.500	6.500	4.583
Wealth and Power	4.571	3.429	2.286
Family Orientation	4.333	3.333	2.333
Moral Self-Discipline	6.000	5.333	4.000
Law-Abiding Conformity	5.000	4.250	3.000
Practical Competence	5.500	4.750	3.750
Public Interest	5.800	5.000	3.600
Relational Affection	5.000	3.333	2.000
Prestige Achievement	4.667	3.333	2.000

Note. The calibration anchors were derived from the 90th, 50th, and 10th percentiles of each variable’s distribution. These anchors represent sample-referenced thresholds rather than externally validated cut points.

**Table 2:** Necessary conditions analysis based on fsQCA.

Antecedent Condition	Consistency	Coverage
WP	0.600	0.610
~WP	0.631	0.639
FO	0.597	0.601
~FO	0.619	0.633
MSD	0.654	0.704
~MSD	0.567	0.544
LAC	0.624	0.657
~LAC	0.612	0.599
PC	0.736	0.702
~PC	0.494	0.535
PI	0.709	0.739
~PI	0.545	0.538
RA	0.664	0.635
~RA	0.559	0.604
PA	0.631	0.611
~PA	0.596	0.635

Note. WP, Wealth and Power; FO, Family Orientation; MSD, Moral Self-Discipline; LAC, Law-Abiding Conformity; PC, Practical Competence; PI, Public Interest; RA, Relational Affection; PA, Prestige Achievement. The tilde “~” denotes set negation, indicating low membership in the corresponding calibrated set.

**Table 3:** Effect sizes and significance testing for necessary conditions.

Condition	Method	Scope	Ceiling Zone	*d*	Accuracy	*p*
WP	CR	0.980	0.000	0.000	100.00%	0.454
	CE	0.980	0.000	0.000	100.00%	0.483
FO	CR	0.980	0.000	0.000	100.00%	0.334
	CE	0.980	0.000	0.000	100.00%	0.354
MSD	CR	0.930	0.000	0.000	99.90%	0.004
	CE	0.930	0.000	0.000	100.00%	0.014
LAC	CR	0.980	0.000	0.000	100.00%	1.000
	CE	0.980	0.000	0.000	100.00%	1.000
PC	CR	0.970	0.002	0.003	99.80%	0.000
	CE	0.970	0.002	0.003	100.00%	0.000
PI	CR	0.960	0.002	0.002	99.80%	0.000
	CE	0.960	0.002	0.002	100.00%	0.000
RA	CR	0.960	0.000	0.000	100.00%	1.000
	CE	0.960	0.000	0.000	100.00%	1.000
PA	CR	0.970	0.000	0.000	100.00%	1.000
	CE	0.970	0.000	0.000	100.00%	1.000

Note. WP, Wealth and Power; FO, Family Orientation; MSD, Moral Self-Discipline; LAC, Law-Abiding Conformity; PC, Practical Competence; PI, Public Interest; RA, Relational Affection; PA, Prestige Achievement. The value of *d* represents the effect size. CR refers to the ceiling regression technique. CE denotes the ceiling envelopment technique. Calibrated fuzzy-set data were used. Permutation tests were based on 1000 resamples.

**Table 4:** NCA analysis of the necessity bottleneck level (%) for a single condition.

AI Literacy	WP	FO	MSD	LAC	PC	PI	RA	PA
0	NN	NN	NN	NN	NN	NN	NN	NN
10	NN	NN	NN	NN	NN	NN	NN	NN
20	NN	NN	NN	NN	NN	NN	NN	NN
30	NN	NN	NN	NN	NN	NN	NN	NN
40	NN	NN	NN	NN	NN	NN	NN	NN
50	NN	NN	NN	NN	NN	NN	NN	NN
60	NN	NN	NN	NN	NN	NN	NN	NN
70	NN	NN	NN	NN	NN	NN	NN	NN
80	NN	NN	NN	NN	NN	NN	NN	NN
90	NN	NN	NN	NN	NN	NN	NN	NN
100	1	1	1.9	NN	8.4	10.9	NN	NN

Note. WP, Wealth and Power; FO, Family Orientation; MSD, Moral Self-Discipline; LAC, Law-Abiding Conformity; PC, Practical Competence; PI, Public Interest; RA, Relational Affection; PA, Prestige Achievement. The ceiling technique that produces the results is CR; NN = not necessary.

**Table 5:** Set-theoretically sufficient configurations associated with membership in the high AI literacy set based on self-reported AILS scores among medical students.

Value Condition	C1	C2	C3	C4
WP	⊖	○	⊖	⊗
FO	⊗	○	⊗	
MSD	●	●	●	●
LAC	⊗	⊗		○
PC		○	○	○
PI	○	○	○	●
RA	⊖	○		●
PA	⊗	○	⊗	
Boolean solution term (*n*)	~WP*~FO*MSD* ~LAC*PI*~RA*~PA (42)	WP*FO*MSD*~LAC* PC*PI*RA*PA (15)	~WP*~FO*MSD* PC*PI*~PA (80)	~WP*MSD*LAC* PC*PI*RA (95)
Raw coverage	0.194	0.170	0.248	0.254
Unique coverage	0.017	0.034	0.025	0.055
Consistency	0.904	0.912	0.908	0.888
Overall solution coverage	0.383			
Overall solution consistency	0.867			

Note. WP, Wealth and Power; FO, Family Orientation; MSD, Moral Self-Discipline; LAC, Law-Abiding Conformity; PC, Practical Competence; PI, Public Interest; RA, Relational Affection; PA, Prestige Achievement. Different symbols indicate different types of conditions. Solid circles (●) indicate core conditions present, hollow circles (○) indicate peripheral conditions present, crossed symbols (⊗) indicate core conditions absent, and dashed symbols (⊖) indicate peripheral conditions absent. Blank cells indicate that the presence or absence of the condition is not specified in the configuration. Boolean solution terms reproduce the intermediate solution. The multiplication sign (*) denotes Boolean conjunction, and the tilde “~” denotes set negation, indicating low membership in the corresponding calibrated set. Values in parentheses are the numbers of cases (*n*) with configuration membership greater than 0.50. These counts are not additive because some cases met this criterion for more than one configuration.

**Table 6:** Robustness checks for the configurational solution under alternative analytical specifications.

Analytical Specification	Number of Configurations	Overall Solution Coverage	Overall Solution Consistency
Primary specification	4	0.383	0.867
Raw consistency threshold of 0.85	4	0.383	0.867
Frequency cutoff of 5	13	0.477	0.848
Frequency cutoff of 15	4	0.365	0.872
PRI threshold of 0.60	5	0.388	0.862
PRI threshold of 0.70	2	0.279	0.898
Calibration at the 85th, 50th, and 15th percentiles	1	0.209	0.885

Note. The primary specification used calibration anchors at the 90th, 50th, and 10th percentiles, a frequency cutoff of 10, a PRI threshold of 0.65, and a raw consistency threshold of 0.80. Each sensitivity analysis varied one analytical specification while holding the remaining parameters constant. Coverage and consistency refer to the overall solution. PRI denotes proportional reduction in inconsistency.

## Data Availability

The data presented in this study are available on request from the corresponding author due to privacy and ethical restrictions.

## Supplementary Materials

The following supporting information can be downloaded at: https://www.mdpi.com/article/10.3390/bs16091458/s1. The supplementary material accompanying this article includes Table S1, Descriptive Statistics and Spearman’s Rank Correlations among Study Variables (N = 1500); Table S2, Complete Truth Table for Membership in the High Perceived AI Literacy Set; Table S3, Complex Solution for Membership in the High Perceived AI Literacy Set; Table S4, Intermediate Solution for Membership in the High Perceived AI Literacy Set; Table S5, Parsimonious Solutions for Membership in the High Perceived AI Literacy Set; and Table S6, Configurations Retained under Each Alternative Analytical Specification.
