# Supplementary material for: Value Configurations Associated with Artificial Intelligence Literacy Among Medical Students: Findings from NCA and fsQCA
Source: Behav Sci (Basel). 2026 Aug 22;16(9):1458. doi: 10.3390/bs16091458 (PMC13603413; doi:10.3390/bs16091458)
Supplement: Supplementary file 1 [file behavsci-16-01458-s001.zip › behavsci-4379617-supplementary.pdf]

Supplementary Materials

Table S1. Descriptive Statistics and Spearman's Rank Correlations among Study Variables (N = 1,500).

Vari-able	*M (SD)*	1	2	3	4	5	6	7	8	9	10	11	12	13	14	15	16	17
1	21.838 (3.082)	1																
2	-	0.157***	1															
3	-	0.846***	0.188***	1														
4	-	0.162***	0.023	-0.154***	1													
5	6.557 (1.484)	-0.011	0.044	0.008	-0.061*	1												
6	6.566 (1.633)	-0.006	-0.022	0.007	-0.047	0.839***	1											
7	6.539 (1.606)	0.030	0.038	0.034	-0.030	0.895***	0.745***	1										
8	6.045 (2.059)	-0.013	0.021	-0.013	-0.001	0.888***	0.676***	0.772***	1									
9	7.078 (1.766)	-0.053*	0.151***	0.001	-0.140***	0.746***	0.465***	0.535***	0.547***	1								
10	3.422 (0.912)	0.023	-0.022	0.002	0.099***	-0.031	0.025	-0.014	0.033	-0.101***	1							
11	3.338 (0.862)	0.160***	-0.150***	0.146***	0.034	-0.040	-0.015	-0.014	-0.011	-0.064*	0.339***	1						
12	4.986 (0.974)	0.015	0.115***	0.046	-0.073**	0.315***	0.236***	0.270***	0.245***	0.340***	0.001	0.221***	1					
13	4.081 (0.868)	0.019	0.030	0.028	0.020	0.113***	0.087***	0.114***	0.125***	0.091***	0.249***	0.383***	0.485***	1				
14	4.681 (0.864)	0.047	0.073**	0.079**	-0.061*	0.368***	0.286***	0.323***	0.325***	0.345***	0.153***	0.251***	0.542***	0.432***	1			
15	4.739 (0.931)	-0.056*	0.248***	0.006	-0.125***	0.422***	0.317***	0.352***	0.320***	0.478***	-0.043	0.039	0.565***	0.316***	0.559***	1		
16	3.489 (1.112)	-0.075**	-0.344***	-0.055*	-0.017	0.121***	0.123***	0.111***	0.137***	0.062*	0.304***	0.415***	0.149***	0.282***	0.270***	0.093***	1	
17	3.396 (1.005)	-0.004	-0.154***	0.011	-0.025	0.003	0.026	-0.008	0.042	-0.019	0.542***	0.466***	0.109***	0.310***	0.256***	0.059*	0.472***	1

Note. M = mean; SD = standard deviation. 1 = age; 2 = gender (0 = male, 1 = female); 3 = academic level (0 = undergraduate, 1 = postgraduate); 4 = year of enrollment (0 = lower year [2025], 1 = higher year [2021-2024]); 5 = AI literacy total score; 6 = Awareness; 7 = Usage; 8 = Evaluation; 9 = Ethics; 10 = Wealth and Power; 11 = Family Orientation; 12 = Moral Self-Discipline; 13 = Law-Abiding Conformity; 14 = Practical Competence; 15 = Public Interest; 16 = Relational Affection; 17 = Prestige Achievement. All coefficients are Spearman's rank correlations and were rounded directly from the full precision estimates. *p < 0.05, **p < 0.01, ***p < 0.001.

Table S2. Complete Truth Table for Membership in the High Perceived AI Literacy Set.

Row	WP	FO	MSD	LAC	PC	PI	RA	PA	Frequency	Raw consistency	PRI	Outcome
1	0	0	0	0	0	0	0	0	82	0.611	0.220	0
2	0	0	0	0	0	0	0	1	10	0.775	0.309	0
3	0	0	0	0	0	0	1	0	10	0.778	0.327	0
4	0	0	0	0	0	0	1	1	7	0.789	0.322	R
5	0	0	0	0	0	1	0	0	11	0.884	0.575	0
6	0	0	0	0	0	1	0	1	3	0.916	0.604	R
7	0	0	0	0	0	1	1	0	1	0.920	0.622	R
8	0	0	0	0	0	1	1	1	5	0.918	0.591	R
9	0	0	0	0	1	0	0	0	12	0.888	0.562	0
10	0	0	0	0	1	0	0	1	4	0.908	0.571	R
11	0	0	0	0	1	0	1	0	5	0.916	0.626	R
12	0	0	0	0	1	0	1	1	2	0.920	0.624	R
13	0	0	0	0	1	1	0	0	9	0.915	0.673	R
14	0	0	0	0	1	1	0	1	2	0.924	0.628	R
15	0	0	0	0	1	1	1	0	9	0.933	0.723	R
16	0	0	0	0	1	1	1	1	3	0.934	0.682	R
17	0	0	0	1	0	0	0	0	4	0.838	0.392	R
18	0	0	0	1	0	0	0	1	0	0.857	0.373	R
19	0	0	0	1	0	0	1	0	4	0.856	0.391	R
20	0	0	0	1	0	0	1	1	1	0.850	0.344	R
21	0	0	0	1	0	1	0	0	3	0.912	0.584	R
22	0	0	0	1	0	1	0	1	1	0.917	0.542	R
23	0	0	0	1	0	1	1	0	0	0.923	0.575	R
24	0	0	0	1	0	1	1	1	3	0.912	0.528	R
25	0	0	0	1	1	0	0	0	1	0.910	0.570	R

Row	WP	FO	MSD	LAC	PC	PI	RA	PA	Frequency	Raw consistency	PRI	Outcome
26	0	0	0	1	1	0	0	1	1	0.911	0.533	R
27	0	0	0	1	1	0	1	0	3	0.911	0.565	R
28	0	0	0	1	1	0	1	1	2	0.911	0.550	R
29	0	0	0	1	1	1	0	0	6	0.923	0.669	R
30	0	0	0	1	1	1	0	1	1	0.922	0.599	R
31	0	0	0	1	1	1	1	0	4	0.934	0.696	R
32	0	0	0	1	1	1	1	1	1	0.929	0.653	R
33	0	0	1	0	0	0	0	0	6	0.899	0.589	R
34	0	0	1	0	0	0	0	1	0	0.922	0.605	R
35	0	0	1	0	0	0	1	0	1	0.927	0.644	R
36	0	0	1	0	0	0	1	1	2	0.932	0.628	R
37	0	0	1	0	0	1	0	0	18	0.915	0.697	1
38	0	0	1	0	0	1	0	1	3	0.936	0.691	R
39	0	0	1	0	0	1	1	0	8	0.935	0.715	R
40	0	0	1	0	0	1	1	1	1	0.939	0.682	R
41	0	0	1	0	1	0	0	0	1	0.919	0.650	R
42	0	0	1	0	1	0	0	1	3	0.933	0.660	R
43	0	0	1	0	1	0	1	0	5	0.944	0.737	R
44	0	0	1	0	1	0	1	1	3	0.942	0.710	R
45	0	0	1	0	1	1	0	0	24	0.923	0.751	1
46	0	0	1	0	1	1	0	1	2	0.938	0.718	R
47	0	0	1	0	1	1	1	0	11	0.942	0.791	1
48	0	0	1	0	1	1	1	1	8	0.943	0.755	R
49	0	0	1	1	0	0	0	0	1	0.901	0.550	R
50	0	0	1	1	0	0	0	1	0	0.916	0.529	R
51	0	0	1	1	0	0	1	0	1	0.917	0.556	R

Row	WP	FO	MSD	LAC	PC	PI	RA	PA	Frequency	Raw consistency	PRI	Outcome
52	0	0	1	1	0	0	1	1	2	0.912	0.506	R
53	0	0	1	1	0	1	0	0	5	0.929	0.705	R
54	0	0	1	1	0	1	0	1	1	0.929	0.618	R
55	0	0	1	1	0	1	1	0	3	0.933	0.662	R
56	0	0	1	1	0	1	1	1	0	0.922	0.592	R
57	0	0	1	1	1	0	0	0	5	0.926	0.655	R
58	0	0	1	1	1	0	0	1	0	0.929	0.617	R
59	0	0	1	1	1	0	1	0	3	0.927	0.661	R
60	0	0	1	1	1	0	1	1	0	0.922	0.626	R
61	0	0	1	1	1	1	0	0	25	0.931	0.764	1
62	0	0	1	1	1	1	0	1	7	0.925	0.676	R
63	0	0	1	1	1	1	1	0	20	0.933	0.772	1
64	0	0	1	1	1	1	1	1	16	0.916	0.699	1
65	0	1	0	0	0	0	0	0	10	0.753	0.253	0
66	0	1	0	0	0	0	0	1	4	0.771	0.246	R
67	0	1	0	0	0	0	1	0	7	0.787	0.299	R
68	0	1	0	0	0	0	1	1	17	0.769	0.278	0
69	0	1	0	0	0	1	0	0	1	0.891	0.474	R
70	0	1	0	0	0	1	0	1	2	0.901	0.469	R
71	0	1	0	0	0	1	1	0	2	0.903	0.532	R
72	0	1	0	0	0	1	1	1	3	0.891	0.467	R
73	0	1	0	0	1	0	0	0	3	0.878	0.460	R
74	0	1	0	0	1	0	0	1	1	0.884	0.451	R
75	0	1	0	0	1	0	1	0	4	0.901	0.557	R
76	0	1	0	0	1	0	1	1	5	0.883	0.507	R
77	0	1	0	0	1	1	0	0	3	0.909	0.565	R

Row	WP	FO	MSD	LAC	PC	PI	RA	PA	Frequency	Raw consistency	PRI	Outcome
78	0	1	0	0	1	1	0	1	2	0.905	0.504	R
79	0	1	0	0	1	1	1	0	7	0.922	0.646	R
80	0	1	0	0	1	1	1	1	5	0.902	0.566	R
81	0	1	0	1	0	0	0	0	5	0.805	0.287	R
82	0	1	0	1	0	0	0	1	0	0.813	0.267	R
83	0	1	0	1	0	0	1	0	4	0.817	0.307	R
84	0	1	0	1	0	0	1	1	5	0.799	0.262	R
85	0	1	0	1	0	1	0	0	0	0.898	0.468	R
86	0	1	0	1	0	1	0	1	0	0.899	0.450	R
87	0	1	0	1	0	1	1	0	1	0.898	0.480	R
88	0	1	0	1	0	1	1	1	2	0.883	0.435	R
89	0	1	0	1	1	0	0	0	1	0.878	0.422	R
90	0	1	0	1	1	0	0	1	1	0.879	0.414	R
91	0	1	0	1	1	0	1	0	1	0.889	0.476	R
92	0	1	0	1	1	0	1	1	4	0.868	0.434	R
93	0	1	0	1	1	1	0	0	2	0.908	0.557	R
94	0	1	0	1	1	1	0	1	1	0.902	0.513	R
95	0	1	0	1	1	1	1	0	1	0.914	0.596	R
96	0	1	0	1	1	1	1	1	11	0.898	0.565	0
97	0	1	1	0	0	0	0	0	2	0.896	0.505	R
98	0	1	1	0	0	0	0	1	0	0.915	0.532	R
99	0	1	1	0	0	0	1	0	0	0.915	0.564	R
100	0	1	1	0	0	0	1	1	2	0.913	0.525	R
101	0	1	1	0	0	1	0	0	4	0.921	0.635	R
102	0	1	1	0	0	1	0	1	2	0.930	0.611	R
103	0	1	1	0	0	1	1	0	3	0.933	0.658	R

Row	WP	FO	MSD	LAC	PC	PI	RA	PA	Frequency	Raw consistency	PRI	Outcome
104	0	1	1	0	0	1	1	1	1	0.925	0.591	R
105	0	1	1	0	1	0	0	0	2	0.917	0.610	R
106	0	1	1	0	1	0	0	1	6	0.923	0.617	R
107	0	1	1	0	1	0	1	0	1	0.933	0.678	R
108	0	1	1	0	1	0	1	1	5	0.925	0.658	R
109	0	1	1	0	1	1	0	0	6	0.930	0.711	R
110	0	1	1	0	1	1	0	1	3	0.931	0.673	R
111	0	1	1	0	1	1	1	0	7	0.944	0.768	R
112	0	1	1	0	1	1	1	1	9	0.927	0.709	R
113	0	1	1	1	0	0	0	0	6	0.869	0.428	R
114	0	1	1	1	0	0	0	1	2	0.886	0.421	R
115	0	1	1	1	0	0	1	0	2	0.886	0.450	R
116	0	1	1	1	0	0	1	1	4	0.870	0.389	R
117	0	1	1	1	0	1	0	0	5	0.912	0.576	R
118	0	1	1	1	0	1	0	1	1	0.917	0.543	R
119	0	1	1	1	0	1	1	0	2	0.917	0.590	R
120	0	1	1	1	0	1	1	1	7	0.901	0.516	R
121	0	1	1	1	1	0	0	0	6	0.887	0.514	R
122	0	1	1	1	1	0	0	1	3	0.899	0.525	R
123	0	1	1	1	1	0	1	0	4	0.907	0.596	R
124	0	1	1	1	1	0	1	1	11	0.885	0.565	0
125	0	1	1	1	1	1	0	0	15	0.909	0.649	0
126	0	1	1	1	1	1	0	1	8	0.915	0.642	R
127	0	1	1	1	1	1	1	0	11	0.920	0.717	1
128	0	1	1	1	1	1	1	1	48	0.896	0.683	1
129	1	0	0	0	0	0	0	0	15	0.782	0.340	0

Row	WP	FO	MSD	LAC	PC	PI	RA	PA	Frequency	Raw consistency	PRI	Outcome
130	1	0	0	0	0	0	0	1	9	0.789	0.322	R
131	1	0	0	0	0	0	1	0	7	0.808	0.351	R
132	1	0	0	0	0	0	1	1	18	0.785	0.325	0
133	1	0	0	0	0	1	0	0	2	0.889	0.460	R
134	1	0	0	0	0	1	0	1	5	0.896	0.483	R
135	1	0	0	0	0	1	1	0	1	0.911	0.496	R
136	1	0	0	0	0	1	1	1	2	0.895	0.479	R
137	1	0	0	0	1	0	0	0	10	0.900	0.554	0
138	1	0	0	0	1	0	0	1	2	0.899	0.521	R
139	1	0	0	0	1	0	1	0	2	0.915	0.600	R
140	1	0	0	0	1	0	1	1	12	0.896	0.576	0
141	1	0	0	0	1	1	0	0	4	0.908	0.571	R
142	1	0	0	0	1	1	0	1	4	0.909	0.544	R
143	1	0	0	0	1	1	1	0	1	0.929	0.625	R
144	1	0	0	0	1	1	1	1	3	0.905	0.570	R
145	1	0	0	1	0	0	0	0	3	0.851	0.337	R
146	1	0	0	1	0	0	0	1	2	0.839	0.312	R
147	1	0	0	1	0	0	1	0	1	0.848	0.321	R
148	1	0	0	1	0	0	1	1	9	0.813	0.303	R
149	1	0	0	1	0	1	0	0	1	0.900	0.464	R
150	1	0	0	1	0	1	0	1	1	0.896	0.448	R
151	1	0	0	1	0	1	1	0	1	0.909	0.482	R
152	1	0	0	1	0	1	1	1	1	0.889	0.446	R
153	1	0	0	1	1	0	0	0	0	0.904	0.512	R
154	1	0	0	1	1	0	0	1	3	0.889	0.464	R
155	1	0	0	1	1	0	1	0	2	0.911	0.537	R

Row	WP	FO	MSD	LAC	PC	PI	RA	PA	Frequency	Raw consistency	PRI	Outcome
156	1	0	0	1	1	0	1	1	6	0.886	0.513	R
157	1	0	0	1	1	1	0	0	3	0.912	0.573	R
158	1	0	0	1	1	1	0	1	6	0.899	0.527	R
159	1	0	0	1	1	1	1	0	4	0.923	0.602	R
160	1	0	0	1	1	1	1	1	9	0.896	0.564	R
161	1	0	1	0	0	0	0	0	0	0.913	0.520	R
162	1	0	1	0	0	0	0	1	0	0.912	0.492	R
163	1	0	1	0	0	0	1	0	0	0.923	0.557	R
164	1	0	1	0	0	0	1	1	1	0.919	0.539	R
165	1	0	1	0	0	1	0	0	5	0.921	0.579	R
166	1	0	1	0	0	1	0	1	3	0.926	0.575	R
167	1	0	1	0	0	1	1	0	2	0.931	0.602	R
168	1	0	1	0	0	1	1	1	2	0.928	0.586	R
169	1	0	1	0	1	0	0	0	1	0.934	0.641	R
170	1	0	1	0	1	0	0	1	2	0.925	0.587	R
171	1	0	1	0	1	0	1	0	2	0.946	0.713	R
172	1	0	1	0	1	0	1	1	3	0.936	0.676	R
173	1	0	1	0	1	1	0	0	2	0.929	0.670	R
174	1	0	1	0	1	1	0	1	5	0.929	0.649	R
175	1	0	1	0	1	1	1	0	6	0.938	0.713	R
176	1	0	1	0	1	1	1	1	2	0.929	0.681	R
177	1	0	1	1	0	0	0	0	2	0.909	0.471	R
178	1	0	1	1	0	0	0	1	0	0.902	0.437	R
179	1	0	1	1	0	0	1	0	1	0.912	0.472	R
180	1	0	1	1	0	0	1	1	2	0.891	0.433	R
181	1	0	1	1	0	1	0	0	2	0.920	0.564	R

Row	WP	FO	MSD	LAC	PC	PI	RA	PA	Frequency	Raw consistency	PRI	Outcome
182	1	0	1	1	0	1	0	1	1	0.909	0.500	R
183	1	0	1	1	0	1	1	0	0	0.928	0.571	R
184	1	0	1	1	0	1	1	1	1	0.900	0.500	R
185	1	0	1	1	1	0	0	0	1	0.921	0.575	R
186	1	0	1	1	1	0	0	1	0	0.909	0.534	R
187	1	0	1	1	1	0	1	0	1	0.929	0.625	R
188	1	0	1	1	1	0	1	1	7	0.907	0.591	R
189	1	0	1	1	1	1	0	0	8	0.911	0.613	R
190	1	0	1	1	1	1	0	1	12	0.904	0.600	0
191	1	0	1	1	1	1	1	0	3	0.930	0.691	R
192	1	0	1	1	1	1	1	1	12	0.896	0.628	0
193	1	1	0	0	0	0	0	0	1	0.782	0.263	R
194	1	1	0	0	0	0	0	1	13	0.757	0.235	0
195	1	1	0	0	0	0	1	0	9	0.793	0.285	R
196	1	1	0	0	0	0	1	1	64	0.705	0.242	0
197	1	1	0	0	0	1	0	0	3	0.891	0.427	R
198	1	1	0	0	0	1	0	1	2	0.885	0.409	R
199	1	1	0	0	0	1	1	0	1	0.901	0.467	R
200	1	1	0	0	0	1	1	1	4	0.874	0.458	R
201	1	1	0	0	1	0	0	0	1	0.892	0.465	R
202	1	1	0	0	1	0	0	1	5	0.880	0.437	R
203	1	1	0	0	1	0	1	0	3	0.900	0.522	R
204	1	1	0	0	1	0	1	1	22	0.859	0.499	0
205	1	1	0	0	1	1	0	0	0	0.908	0.519	R
206	1	1	0	0	1	1	0	1	2	0.899	0.490	R
207	1	1	0	0	1	1	1	0	3	0.920	0.589	R

Row	WP	FO	MSD	LAC	PC	PI	RA	PA	Frequency	Raw consistency	PRI	Outcome
208	1	1	0	0	1	1	1	1	13	0.890	0.589	0
209	1	1	0	1	0	0	0	0	2	0.816	0.261	R
210	1	1	0	1	0	0	0	1	6	0.791	0.223	R
211	1	1	0	1	0	0	1	0	2	0.811	0.248	R
212	1	1	0	1	0	0	1	1	27	0.738	0.223	0
213	1	1	0	1	0	1	0	0	2	0.887	0.397	R
214	1	1	0	1	0	1	0	1	3	0.881	0.396	R
215	1	1	0	1	0	1	1	0	1	0.889	0.405	R
216	1	1	0	1	0	1	1	1	4	0.853	0.406	R
217	1	1	0	1	1	0	0	0	1	0.882	0.420	R
218	1	1	0	1	1	0	0	1	1	0.865	0.381	R
219	1	1	0	1	1	0	1	0	1	0.888	0.448	R
220	1	1	0	1	1	0	1	1	31	0.825	0.430	0
221	1	1	0	1	1	1	0	0	2	0.902	0.509	R
222	1	1	0	1	1	1	0	1	8	0.885	0.498	R
223	1	1	0	1	1	1	1	0	1	0.913	0.568	R
224	1	1	0	1	1	1	1	1	31	0.859	0.551	0
225	1	1	1	0	0	0	0	0	1	0.903	0.457	R
226	1	1	1	0	0	0	0	1	2	0.905	0.446	R
227	1	1	1	0	0	0	1	0	1	0.917	0.505	R
228	1	1	1	0	0	0	1	1	4	0.885	0.441	R
229	1	1	1	0	0	1	0	0	0	0.921	0.542	R
230	1	1	1	0	0	1	0	1	1	0.919	0.525	R
231	1	1	1	0	0	1	1	0	0	0.934	0.582	R
232	1	1	1	0	0	1	1	1	5	0.905	0.533	R
233	1	1	1	0	1	0	0	0	3	0.922	0.571	R

Row	WP	FO	MSD	LAC	PC	PI	RA	PA	Frequency	Raw consistency	PRI	Outcome
234	1	1	1	0	1	0	0	1	2	0.912	0.540	R
235	1	1	1	0	1	0	1	0	3	0.936	0.645	R
236	1	1	1	0	1	0	1	1	9	0.908	0.609	R
237	1	1	1	0	1	1	0	0	2	0.923	0.624	R
238	1	1	1	0	1	1	0	1	8	0.912	0.586	R
239	1	1	1	0	1	1	1	0	3	0.940	0.701	R
240	1	1	1	0	1	1	1	1	15	0.912	0.677	1
241	1	1	1	1	0	0	0	0	1	0.884	0.400	R
242	1	1	1	1	0	0	0	1	3	0.878	0.376	R
243	1	1	1	1	0	0	1	0	3	0.885	0.409	R
244	1	1	1	1	0	0	1	1	15	0.833	0.346	0
245	1	1	1	1	0	1	0	0	1	0.911	0.506	R
246	1	1	1	1	0	1	0	1	2	0.900	0.468	R
247	1	1	1	1	0	1	1	0	2	0.916	0.538	R
248	1	1	1	1	0	1	1	1	10	0.870	0.463	0
249	1	1	1	1	1	0	0	0	2	0.892	0.487	R
250	1	1	1	1	1	0	0	1	9	0.886	0.491	R
251	1	1	1	1	1	0	1	0	3	0.906	0.557	R
252	1	1	1	1	1	0	1	1	40	0.829	0.480	0
253	1	1	1	1	1	1	0	0	6	0.906	0.598	R
254	1	1	1	1	1	1	0	1	17	0.891	0.585	0
255	1	1	1	1	1	1	1	0	8	0.918	0.675	R
256	1	1	1	1	1	1	1	1	114	0.847	0.626	0

Note. WP = Wealth and Power; FO = Family Orientation; MSD = Moral Self-Discipline; LAC = Law-Abiding Conformity; PC = Practical Competence; PI = Public Interest; RA = Relational Affection; PA = Prestige Achievement. A value of 1 denotes presence and 0 denotes absence in the corresponding truth table row. Frequency is the number of cases assigned to each row using the 0.50 crossover. Outcome 1 denotes a retained sufficient row, Outcome 0 denotes an empirically observed row that was not retained, and R denotes a logical remainder because the row frequency was below 10. The primary analysis used a frequency cutoff of 10, a raw consistency threshold of 0.80, and a PRI threshold of 0.65. The effective raw consistency cutoff among the retained rows was 0.896.

Table S3. Complex Solution for Membership in the High Perceived AI Literacy Set.

Configuration	Boolean solution term	Raw coverage	Unique coverage	Consistency
C1	~WP*~FO*MSD*~LAC*PI*~RA*~PA	0.194	0.017	0.904
C2	WP*FO*MSD*~LAC*PC*PI*RA*PA	0.170	0.034	0.912
C3	~WP*~FO*MSD*PC*PI*~PA	0.248	0.025	0.908
C4	~WP*MSD*LAC*PC*PI*RA	0.254	0.055	0.888
Overall solution		0.383	—	0.867

Note. WP = Wealth and Power; FO = Family Orientation; MSD = Moral Self-Discipline; LAC = Law-Abiding Conformity; PC = Practical Competence; PI = Public Interest; RA = Relational Affection; PA = Prestige Achievement. The multiplication sign (*) denotes Boolean conjunction, and the tilde (~) denotes set negation. Logical remainders were excluded from the complex solution.

Table S4. Intermediate Solution for Membership in the High Perceived AI Literacy Set.

Configuration	Boolean solution term	Raw coverage	Unique coverage	Consistency
C1	~WP*~FO*MSD*~LAC*PI*~RA*~PA	0.194	0.017	0.904
C2	WP*FO*MSD*~LAC*PC*PI*RA*PA	0.170	0.034	0.912
C3	~WP*~FO*MSD*PC*PI*~PA	0.248	0.025	0.908
C4	~WP*MSD*LAC*PC*PI*RA	0.254	0.055	0.888
Overall solution		0.383	—	0.867

Note. WP = Wealth and Power; FO = Family Orientation; MSD = Moral Self-Discipline; LAC = Law-Abiding Conformity; PC = Practical Competence; PI = Public Interest; RA = Relational Affection; PA = Prestige Achievement. The multiplication sign (*) denotes Boolean conjunction, and the tilde (~) denotes set negation. No directional expectations were specified, and no logical remainder was treated as an easy counterfactual. Under this specification, the intermediate solution was identical to the complex solution.

Table S5. Parsimonious Solutions for Membership in the High Perceived AI Literacy Set.

Model	Boolean solution term	Raw coverage	Unique coverage	Consistency
M1	MSD*~LAC	0.396	0.115	0.815
M1	~FO*LAC*~PA	0.312	0.085	0.819
M1	~WP*MSD*PI*RA	0.308	0.048	0.866
M1	Overall solution	0.560	—	0.778
M2 (reference)	MSD*~LAC	0.396	0.071	0.815
M2 (reference)	~FO*MSD*~PA	0.338	0.038	0.838
M2 (reference)	~WP*MSD*PI*RA	0.308	0.046	0.866
M2 (reference)	Overall solution	0.514	—	0.796
M3	MSD*~LAC	0.396	0.127	0.815
M3	~WP*~FO*LAC	0.315	0.075	0.823
M3	~WP*MSD*PI*RA	0.308	0.037	0.866
M3	Overall solution	0.550	—	0.781
M4	MSD*~LAC	0.396	0.091	0.815
M4	~WP*~FO*MSD	0.343	0.033	0.839
M4	~WP*MSD*PI*RA	0.308	0.035	0.866
M4	Overall solution	0.508	—	0.799

Note. WP = Wealth and Power; FO = Family Orientation; MSD = Moral Self-Discipline; LAC = Law-Abiding Conformity; PC = Practical Competence; PI = Public Interest; RA = Relational Affection; PA = Prestige Achievement. The multiplication sign (*) denotes Boolean conjunction, and the tilde (~) denotes set negation. Logical remainders were permitted without directional restrictions. Exact Boolean minimization yielded four equally parsimonious models. Model M2 was selected in the fsQCA 4.1 prime implicant chart as the reference model for classifying core and peripheral conditions. Conditions shared by an intermediate configuration and the corresponding M2 term or terms were classified as core; the remaining conditions in the intermediate configuration were classified as peripheral. For C1, two M2 terms corresponded to the intermediate configuration, and the shared literals from both terms were retained as core conditions. The other equally parsimonious models are reported to make the model ambiguity transparent. Raw and unique coverage are reported for each solution term; the final row for each model reports overall solution coverage and consistency.

Table S6. Configurations Retained under Each Alternative Analytical Specification.

Analytical specification	Configuration	WP	FO	MSD	LAC	PC	PI	RA	PA	Boolean solution term	Counterpart in Table 5	Raw coverage	Unique coverage	Consistency
Primary specification	C1	⊖	⊗	●	⊗		○	⊖	⊗	~WP*~FO*MSD*~LAC*PI*~RA*~PA	C1	0.194	0.017	0.904
	C2	○	○	●	⊗	○	○	○	○	WP*FO*MSD*~LAC*PC*PI*RA*PA	C2	0.170	0.034	0.912
	C3	⊗	⊗	●		○	○		⊗	~WP*~FO*MSD*PC*PI*~PA	C3	0.248	0.025	0.908
	C4	⊗		●	○	○	●	●		~WP*MSD*LAC*PC*PI*RA	C4	0.254	0.055	0.888
	Overall solution											*0.383*	—	*0.867*
Raw consistency threshold of 0.85	C1	⊖	⊗	●	⊗		○	⊖	⊗	~WP*~FO*MSD*~LAC*PI*~RA*~PA	C1	0.194	0.017	0.904
	C2	○	○	●	⊗	○	○	○	○	WP*FO*MSD*~LAC*PC*PI*RA*PA	C2	0.170	0.034	0.912
	C3	⊖	⊗	●		○	○		⊗	~WP*~FO*MSD*PC*PI*~PA	C3	0.248	0.025	0.908
	C4	⊗		●	○	○	●	●		~WP*MSD*LAC*PC*PI*RA	C4	0.254	0.055	0.888
	Overall solution											*0.383*	—	*0.867*
Frequency cutoff of 5	C1	⊗		●		●	●	●		~WP*MSD*PC*PI*RA	—	0.290	0.025	0.879
	C2	⊗	⊗	○	●	●	●			~WP*~FO*MSD*LAC*PC*PI	—	0.238	0.006	0.906
	C3	⊗	⊗			●	●	⊖	⊖	~WP*~FO*PC*PI*~RA*~PA	—	0.236	0.004	0.895
	C4	⊖	⊖	○			○	⊖	⊖	~WP*~FO*MSD*PI*~RA*~PA	—	0.233	0.006	0.899
	C5	⊗	⊗		⊖	●	●		⊖	~WP*~FO*~LAC*PC*PI*~PA	—	0.230	0.007	0.902
	C6	⊗		●	⊗	○	●		⊖	~WP*MSD*~LAC*PC*PI*~PA	—	0.227	0.005	0.913
	C7	⊗	⊖	●	⊗		●		⊖	~WP*~FO*MSD*~LAC*PI*~PA	—	0.224	0.006	0.903
	C8		○	●	○	○	○	●	⊗	FO*MSD*LAC*PC*PI*RA*~PA	—	0.193	0.009	0.909
	C9		○	●	⊗	●	●	●	○	FO*MSD*~LAC*PC*PI*RA*PA	—	0.189	0.018	0.905
	C10	⊗	⊗	○	●	○		⊖	⊖	~WP*~FO*MSD*LAC*PC*~RA*~PA	—	0.188	0.003	0.921
	C11		⊖	●	⊗	●	●	●	⊗	~FO*MSD*~LAC*PC*PI*RA*~PA	—	0.174	0.005	0.934
	C12	⊗	○	●	⊗	○		●	○	~WP*FO*MSD*~LAC*PC*RA*PA	—	0.168	0.005	0.918
	C13	⊗	⊖	●	⊗	○		●	⊗	~WP*~FO*MSD*~LAC*PC*RA*~PA	—	0.166	0.003	0.936
	Overall solution											*0.477*	—	*0.848*
Frequency cutoff of 15	C1	⊖	⊗	●	⊗		●	⊖	⊖	~WP*~FO*MSD*~LAC*PI*~RA*~PA	C1	0.194	0.041	0.904
	C2	○	○	●	⊗	○	○	○	○	WP*FO*MSD*~LAC*PC*PI*RA*PA	C2	0.170	0.035	0.912
	C3	⊗		○	○	○	●	●	○	~WP*MSD*LAC*PC*PI*RA*PA	—	0.217	0.046	0.892
	C4	⊖	⊗	○	○	○	●		⊖	~WP*~FO*MSD*LAC*PC*PI*~PA	—	0.209	0.030	0.925
	Overall solution											*0.365*	—	*0.872*
PRI threshold of 0.60	C1	⊗	⊖	●	⊗		●	⊖	⊖	~WP*~FO*MSD*~LAC*PI*~RA*~PA	C1	0.194	0.017	0.904
	C2	○	○	●	⊗	○	○	○	○	WP*FO*MSD*~LAC*PC*PI*RA*PA	C2	0.170	0.034	0.912
	C3	⊗	⊖	●		○	●		⊖	~WP*~FO*MSD*PC*PI*~PA	C3	0.248	0.010	0.908
	C4	⊗		●	○	○	●	○		~WP*MSD*LAC*PC*PI*RA	C4	0.254	0.036	0.888
	C5	⊗		●	○	○	●		⊖	~WP*MSD*LAC*PC*PI*~PA	—	0.245	0.005	0.902

	Overall solution											*0.388*	—	*0.862*
PRI threshold of 0.70	C1	⊖	⊗	○		●	●		⊗	~WP*~FO*MSD*PC*PI*~PA	C3	0.248	0.079	0.908
	C2	⊖		○	○	○	●	●	⊗	~WP*MSD*LAC*PC*PI*RA*~PA	—	0.200	0.031	0.915
	Overall solution											*0.279*	—	*0.898*

Note. WP = Wealth and Power; FO = Family Orientation; MSD = Moral Self-Discipline; LAC = Law-Abiding Conformity; PC = Practical Competence; PI = Public Interest; RA = Relational Affection; PA = Prestige Achievement. Different symbols indicate different types of conditions. Solid circles (●) indicate core conditions present, hollow circles (○) indicate peripheral conditions present, crossed symbols (⊗) indicate core conditions absent, and dashed symbols (⊖) indicate peripheral conditions absent. Blank cells indicate that the presence or absence of the condition is not specified in the configuration. Core and peripheral status was assigned by comparing the intermediate and parsimonious solutions. The calibration-robustness specification reported in Table 6 is omitted here because it recalibrates all variables onto different fuzzy sets.
